# Interpretable predictions of chaotic dynamical systems using dynamical system deep learning

**DOI:** 10.1038/s41598-024-53169-y

**Published:** 2024-02-07

**Authors:** Mingyu Wang, Jianping Li

**Affiliations:** 1 Frontiers Science Center for Deep Ocean Multi-Spheres and Earth System (FDOMES)/Key Laboratory of Physical Oceanography/Academy of Future Ocean/Center for Ocean Carbon Neutrality, Ocean University of China, Qingdao, 266100 China; 2 Laoshan Laboratory, Qingdao, 266237 China

**Keywords:** Nonlinear phenomena, Atmospheric dynamics, Applied mathematics

## Abstract

Making accurate predictions of chaotic dynamical systems is an essential but challenging task with many practical applications in various disciplines. However, the current dynamical methods can only provide short-term precise predictions, while prevailing deep learning techniques with better performances always suffer from model complexity and interpretability. Here, we propose a new dynamic-based deep learning method, namely the dynamical system deep learning (DSDL), to achieve interpretable long-term precise predictions by the combination of nonlinear dynamics theory and deep learning methods. As validated by four chaotic dynamical systems with different complexities, the DSDL framework significantly outperforms other dynamical and deep learning methods. Furthermore, the DSDL also reduces the model complexity and realizes the model transparency to make it more interpretable. We firmly believe that the DSDL framework is a promising and effective method for comprehending and predicting chaotic dynamical systems.

## Introduction

Complex, nonlinear dynamical systems are almost ubiquitous in the natural and human world, such as climate systems, ecosystems and financial systems^1–3^. Centuries-old efforts to comprehend and predict such systems have spurred developments in various fields, but have been hindered by their chaotic behaviors^4^, which makes it exceptionally difficult to achieve long-term precise predictions^5^. Studies revealed that the chaotic time series generated by any variable contains abundant dynamical information of the whole system^5,6^. How to exploit the information hidden in the time series data and establish an effective prediction model to accurately predict the future as long as possible, is of great importance in many disciplines^7–9^.

After decades of research, various methods have been proposed to reconstruct dynamics and make predictions of chaotic dynamical systems, and the phase-space reconstruction is undoubtedly one of the most representative dynamical methods. The Takens embedding theorem^10–12^ shows how delayed-coordinates of a single time series can be used as proxy variables to reconstruct dynamics for the underlying deterministic process. Sauer et al.^13^ and Deyle et al.^14^ further generalized the delayed embedding theorem and demonstrated that multivariate time series can also be used in reconstructing dynamics. Ma et al.^15^ proposed an inverse delayed embedding (IDE) method, which is the inverse implementation of the delayed embedding reconstruction. Furthermore, studies^16,17^ combined the delayed embedding theorem with the generalized embedding theorem, but only consider the linear relationships of various factors while ignore the nonlinear interactions among them.

In recent years, with rapid developments in computing power and algorithmic innovations of deep learning techniques, more and more studies have applied various deep learning methods to predictions of chaotic time series, such as long short-term memory network^18–20^, reservoir computing^20–22^, residual network^23,24^, anticipated learning machine^25^, etc. Despite good performances due to the ability of considering nonlinear interactions among variables, deep learning techniques have always been called the “black-box”, which leads to deep learning models being untrusted in some key areas for the lack of model interpretability^26,27^. In addition, the era of big data has witnessed a rapid accumulation of various data, and samples with massive size and high dimensionality pose unique computational and statistical challenges for deep learning methods^28,29^.

Here, we propose a new dynamics-based deep learning method, namely the dynamical system deep learning (DSDL), combining dynamical methods with deep learning methods. Several experiments on four chaotic dynamical systems with different complexities have significantly demonstrated the superior performances of the DSDL over other dynamical and deep learning methods used for comparison in this work. Despite the trade-off between model interpretability and performance^30^, the DSDL not only greatly improves the model performance, but also realizes the model transparency to make it more interpretable.

### DSDL framework

**Figure 1:**
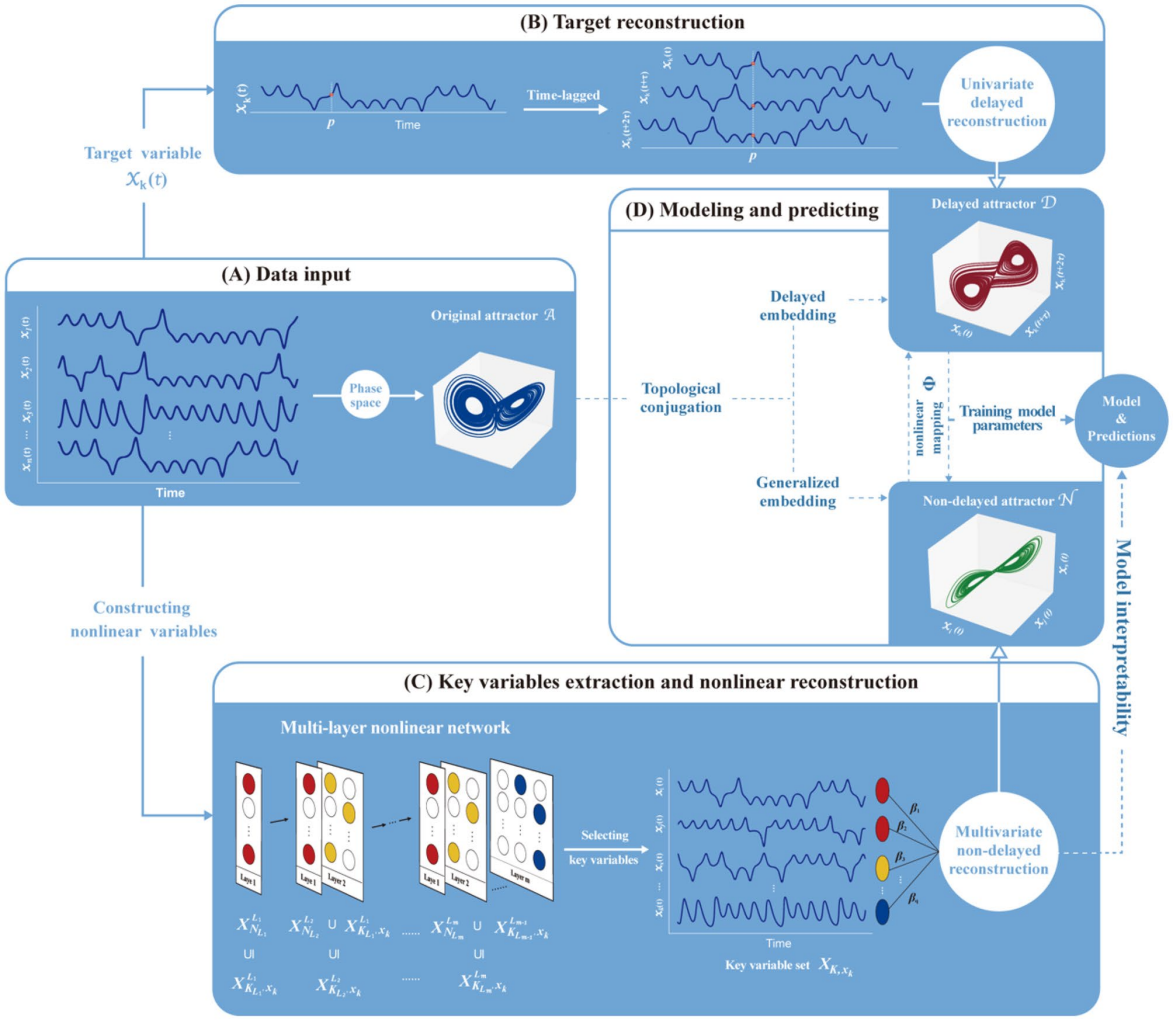
Architecture of the DSDL framework. (**A**) The input data is constructed by n-dimensional time series data $x_{i} \left( t \right), i = 1, 2, \ldots , n$ of one chaotic dynamical system, which has an original attractor $\mathbf {A}$. (**B**) After selecting one time series as the target variable $x_{k} \left( t \right)$, $k \in \left[ {1,n} \right]$, we can reconstruct a delayed attractor $\mathbf {D}$ based on the time-lagged coordinates of $x_{k} \left( t \right)$ with suitable embedding dimension and time delay. (**C**) To take full advantage of the nonlinear interactions among variables, we construct a multi-layer nonlinear network and we can get the candidate variable set ${\varvec{X}}_{{\varvec{N}}}$. From ${\varvec{X}}_{{\varvec{N}}}$, we can select the key variable set ${\varvec{X}}_{{{\varvec{K}},\user2{ x}_{{\varvec{k}}} }}$ of the target variable $x_{k} \left( t \right)$ though the CVSR method to reconstruct a non-delayed attractor $\mathbf {N}$. (**D**) Based on the embedding theorems, both reconstructed attracts ($\mathbf {D}, \mathbf {N}$) are topologically conjugated to the original one ($\mathbf {A}$), so there is a diffeomorphism map ${ }{{\varvec{\Phi}}}:\mathbf {N} \to \mathbf {D}$. Then we can obtain the DSDL prediction model for the target variable $x_{k} \left( t \right)$. using the corresponding training set to fit ${{\varvec{\Phi}}}$.

As particularly shown in Fig. 1A, with $n$ dimensional time series data $x_{i} \left( t \right), i = 1,{ }2,{ } \ldots ,{ }n$, of one chaotic dynamical system (we name it as the target system), every time series can be inputted as one primitive system variable into the DSDL framework, and all primitive system variables constitute the primitive variable set ${\varvec{X}}$, ${\varvec{X}} = \{ x_{1} ,x_{2} , \ldots ,x_{n} \} .$ In a chaotic dynamical system, due to dissipation, the steady dynamics after a transient phase is generally constrained into a subspace^31^, which is the attractor (${\mathbf{A}}$) of the target system. The phase-space technique^10–14^ makes it possible to have two different dynamical methods of reconstructing an attractor of the target system, which are respectively named as the univariate and multivariate way.

The univariate way (Fig. 1B), according to the delayed embedding theory^10–12^, is reconstructed by the time-lagged coordinates of a single variable $x_{k} \left( t \right)$, $k \in \left[ {1, n} \right]$ (we name it as the target variable), thus we can get a “delayed attractor” in the form of $\mathbf {D}$($x_{k} \left( t \right),{ }x_{k} \left( {t + \tau } \right),x_{k} \left( {t + 2\tau } \right), \ldots$) with suitable embedding dimension $L$ and time delay interval $\tau$. This delayed attractor $\mathbf {D}$ is aimed to obtain the temporal information of the target variable^16^. For the DSDL framework, in order to establish a complete prediction model for the target system, every primitive system variable needs to be used as the target variable, that is to say, we must separately establish the DSDL model for each primitive system variable.

The multivariate way (Fig. 1C), based on the generalized embedding theorem^13–15^, is reconstructed by multiple variables and we can get a “non-delayed attractor” in the form of $\bf {N}$($x_{i} \left( t \right),{ }x_{j} \left( t \right),x_{s} \left( t \right), \ldots$), $i,{ }j, s, \ldots \in \left[ {1, n} \right]$. This non-delayed attractor $\mathbf {N}$ is aimed to get the spatial information among system variables^14^. Ma et al.^16^ randomly chose index tuple $l = \left( {i,{ }j, s, \ldots } \right)$ from any combinations of primitive system variables**,** which only consider the linear relationships among them. To take full advantage of the nonlinear interactions among system variables, we construct a multi-layer nonlinear network in the DSDL framework and get the candidate variable set ${\varvec{X}}_{{\varvec{N}}}$, ${\varvec{X}}_{{{\varvec{N}}_{{L_{i} }} }}^{{L_{i} }} \subset {\varvec{X}}_{{\varvec{N}}} { }\left( {i \in \left[ {1, m} \right]} \right)$, where $m$ represents the number of layers in the network, $L_{i}$ denotes the *i*th layer, ${\varvec{N}}_{{L_{i} }}$ is the sample size of this layer, and ${\varvec{X}}_{{{\varvec{N}}_{{L_{i} }} }}^{{L_{i} }}$ is the candidate variable subset of the *i*th layer constructed by all monomials of *i*th-order based on ${\varvec{X}},$ when $i \ge 2$ the monomials are nonlinear. However, we stress that not all variables in the set ${\varvec{X}}_{{\varvec{N}}}$ have a positive impact on predicting the target variable $x_{k} \left( t \right)$, thus we need to select those variables that truly control the evolution of the target variable from ${\varvec{X}}_{{\varvec{N}}}$ to construct the key variables set ${\varvec{X}}_{{{\varvec{K}},\user2{ x}_{{\varvec{k}}} }}$ of $x_{k} \left( t \right)$ by using the cross-validation-based stepwise regression^32^ (CVSR) method. In this way, we can efficiently explore the crucial information and remove redundant information for the DSDL model to reduce model complexity. Thus the non-delayed attractor $\mathbf {N}$ in the DSDL framework is constructed by those key variables we selected.

How to determine the embedding dimension $L$ and time delay $\tau$ is an important topic in the state space reconstruction process, and several criteria have been proposed to the time series^33^. From the embedding theorems^10–14^, we must ensure that the dimension $L$ for reconstructing the above attractors ($\mathbf {D}$, $\mathbf {N}$) is large enough, i.e. $L > 2d_{A}$ where $d_{A}$ is the box counting dimension of the attractor, and let $\tau$ be a positive time interval. Here, we use the False Nearest Neighbors^34^ (FNNs) method to determine the minimal embedding dimension and simply set $\tau$ as one lag in the time series. However, we find that the dimension $L$ of our DSDL model (also the number of key variables selected, as shown in Table S1) is usually much larger than the minimal embedding dimension, which meets the requirement of reconstruction.

Ultimately, since both reconstructed attractors are topologically conjugated to the original attractor, there is a diffeomorphism map between them, that is, ${{\varvec{\Phi}}}:\mathbf {N} \to \mathbf {D}$^13^ (Fig. S1). On this basis, we can obtain the DSDL prediction model for the target variable $x_{k} \left( t \right)$, with several parameters to be determined, in the form of

$$x_{k} \left( {t + \tau } \right) = \varphi \left( {x_{i} \left( t \right),{ }x_{j} \left( t \right),x_{s} \left( t \right), \ldots } \right).$$

Then, we can use the corresponding training data set to fit $\varphi$ and train the model parameters (Fig. 1D). Unlike the statistical model, the DSDL model is more similar to the dynamical model, which is aimed to exploit the dynamical equations/operators to achieve successive predictions using one model, instead of getting discrete predictions using several statistical models.

## Results

### Model performances of DSDL in four chaotic dynamical systems with different complexities

Firstly, our proposed method is tested on three chaotic dynamical systems with different complexities to demonstrate its effectiveness and robustness, including the 3-variable Lorenz system^35^, 4-variable hyperchaotic Lorenz system^36^, 5-variable conceptual ocean–atmosphere coupled Lorenz system^37^ (SI Appendix). Among them, the first two systems are autonomous systems, and the 5-dimensional coupled Lorenz system belongs to a nonautonomous system. With multiple time series data outputted by these systems, sources of predictability are believed to come from the temporal and spatial information hidden in those time series^15^. Here, prediction results of linear models (A linear model refers to a prediction model established solely using the primitive system variables themselves and without nonlinear monomials) and DSDL models are compared in all three systems (Figs. S2, 2).

**Figure 2:**
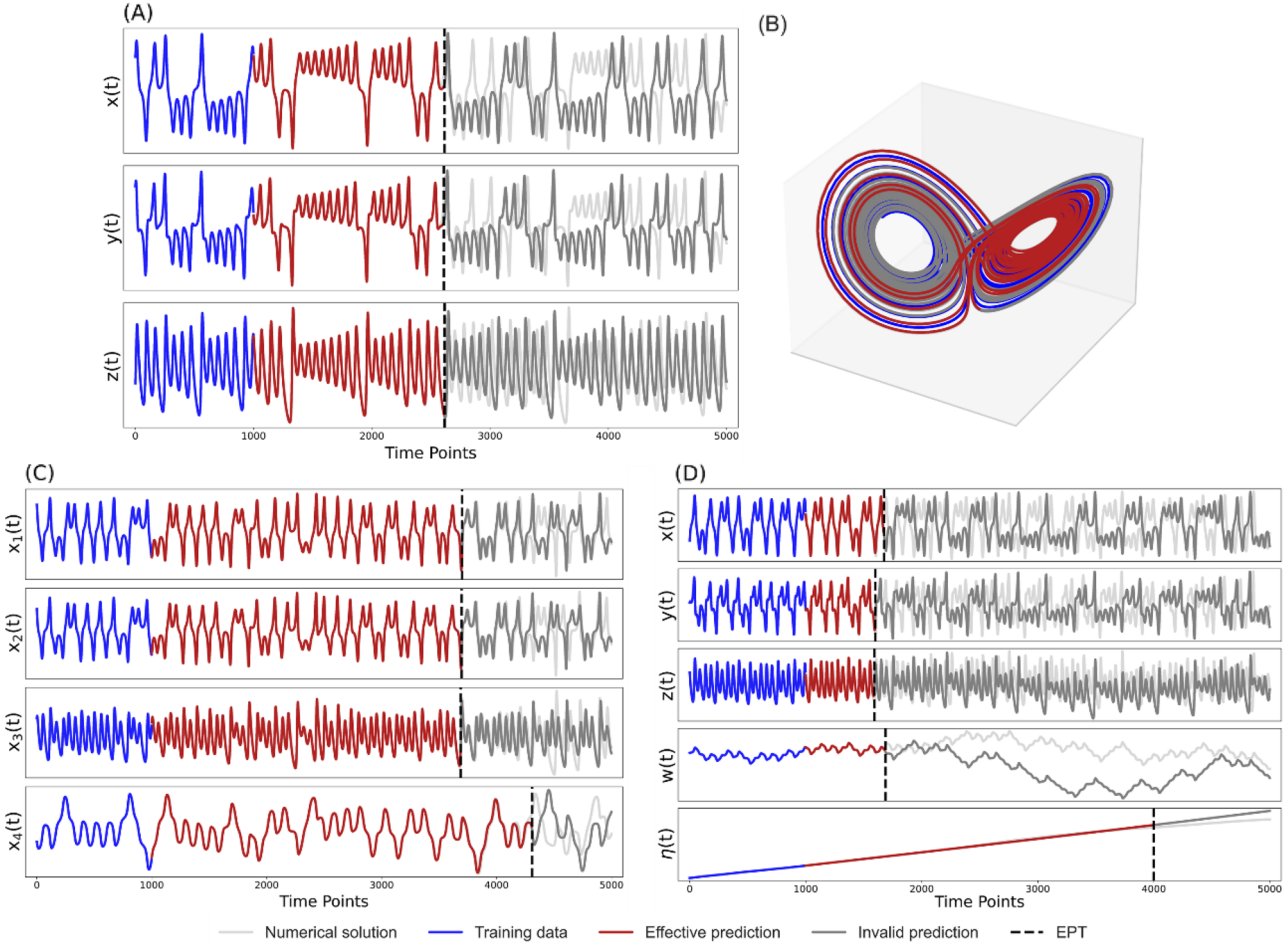
Prediction results of DSDL models in three different chaotic dynamical systems. (**A**) The prediction series of the Lorenz system. The light grey line shows the numerical solutions (true state), the blue line shows the training set, the red line represents the effective predictions and the dark grey line represents the invalid predictions in the corresponding test set. The vertical black dashed line marks the effective prediction time (EPT). Using a training set of 10^4^ time points, only the last 10^3^ time points are shown in this figure. (**B**) The prediction trajectory of the Lorenz attractor. (**C**) Same as (**A**), but for the hyperchaotic Lorenz system. (**D**) Same as (**A**), but for the conceptual ocean–atmosphere coupled Lorenz system.

As a paradigmatic chaotic dynamical system, the Lorenz system outputs three primitive system variables with obvious chaotic oscillations under certain parameter requirements. However, prediction series of the linear model only exhibit nonlinear characteristics for a very short period of time and converge quickly to a fixed point with basically no effective predictions (Fig. S2A). Besides, the prediction trajectory is always off the Lorenz attractor (Fig. S2B), which indicates that we cannot effectively reconstruct the dynamics by only considering the linear relationships among system variables. Using the same training/test sets as the linear model, the prediction series of the DSDL model always maintains the chaotic characteristics of the Lorenz system, accompanied by a significant improvement of effective prediction time (EPT, Fig. 2A). More importantly, the prediction trajectory is consistently on the Lorenz attractor (Fig. 2B), which demonstrates that the DSDL model is able to successfully reconstruct the nonlinear dynamics of the target system. Similar results are also found in the hyperchaotic Lorenz system and the conceptual ocean–atmosphere coupled Lorenz system (Figs. 2C, D, S2C,  D).

Furthermore, we note that the EPTs of different variables in one system are not always the same (Fig. 2C, D). For example, the EPT of variable $\eta$ in the conceptual coupled Lorenz system, representing the ocean pycnocline to simulate features of the slow-changing deep ocean, is much longer than other variables. This further indicates that prediction results of DSDL models are highly consistent with the actual physical properties and predictabilities of various variables in different dynamical processes.

**Figure 3:**
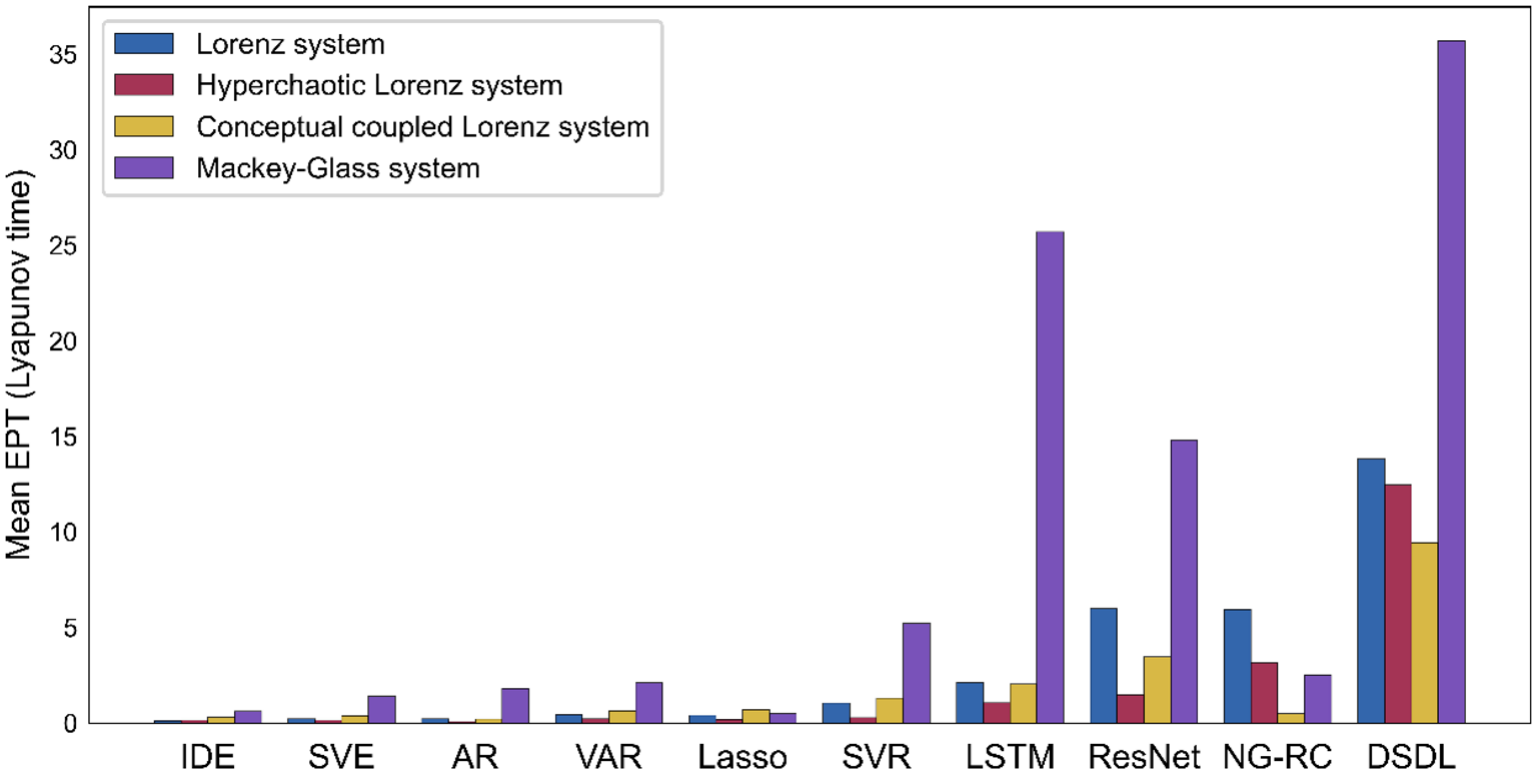
Comparisons between the DSDL and other existing dynamical and machine learning methods in four different chaotic dynamical systems. Using the mean EPT (Lyapunov time), we compare the model predictive capabilities of different methods in four chaotic dynamical systems. The mean EPT is obtained by 100 different training/test sets, and the higher the mean EPT, the better the method performs.

Using the mean EPT (normalized by the Lyapunov time) of 100 different training/test sets to quantify the model predictive capability, we compare the DSDL with nine existing dynamical and machine learning methods used for predicting chaotic time series (SI Appendix). In order to make the results more rigorous, we also incorporate the Mackey–Glass equation^38^ for comparison, which is a nonlinear time delay differential equation and has a completely different construction from the three systems mentioned above. Clearly, the DSDL method shows the best predictive performances in all four chaotic dynamical systems, and is much ahead of other popular deep learning methods (Fig. 3). Gauthier et al.^39^ proposed the next generation reservoir computing (NG-RC) method, which has certain similarities with DSDL. However, the NG-RC method can accurately predict approximately ~ 6 Lyapunov time for the $x$ variable of the Lorenz system, while the DSDL method can predict about ~ 14 Lyapunov time. Moreover, the modeling time of the DSDL (about 2 min for the Lorenz system) is significantly shorter than most of the methods, which further demonstrates the superiority of the DSDL method.

### Interpretability of DSDL models

Traditional deep learning models have achieved remarkable performances in many important domains^40^. However, it is often difficult to explain the prediction results due to their over-parameterized “black-box” nature and lack of interpretability^41–43^. The opposite of “black-box” is transparency, and transparent models convey some degree of *ante-hoc* interpretability by themselves^44^. As a dynamics-based deep learning method, all processes of the DSDL framework are clearly visible during the establishment. And we can further explore and clarify the roles of various components in the DSDL model when we break down a complete DSDL model for one target variable into layers.

#### Chaotic characteristics

**Figure 4:**
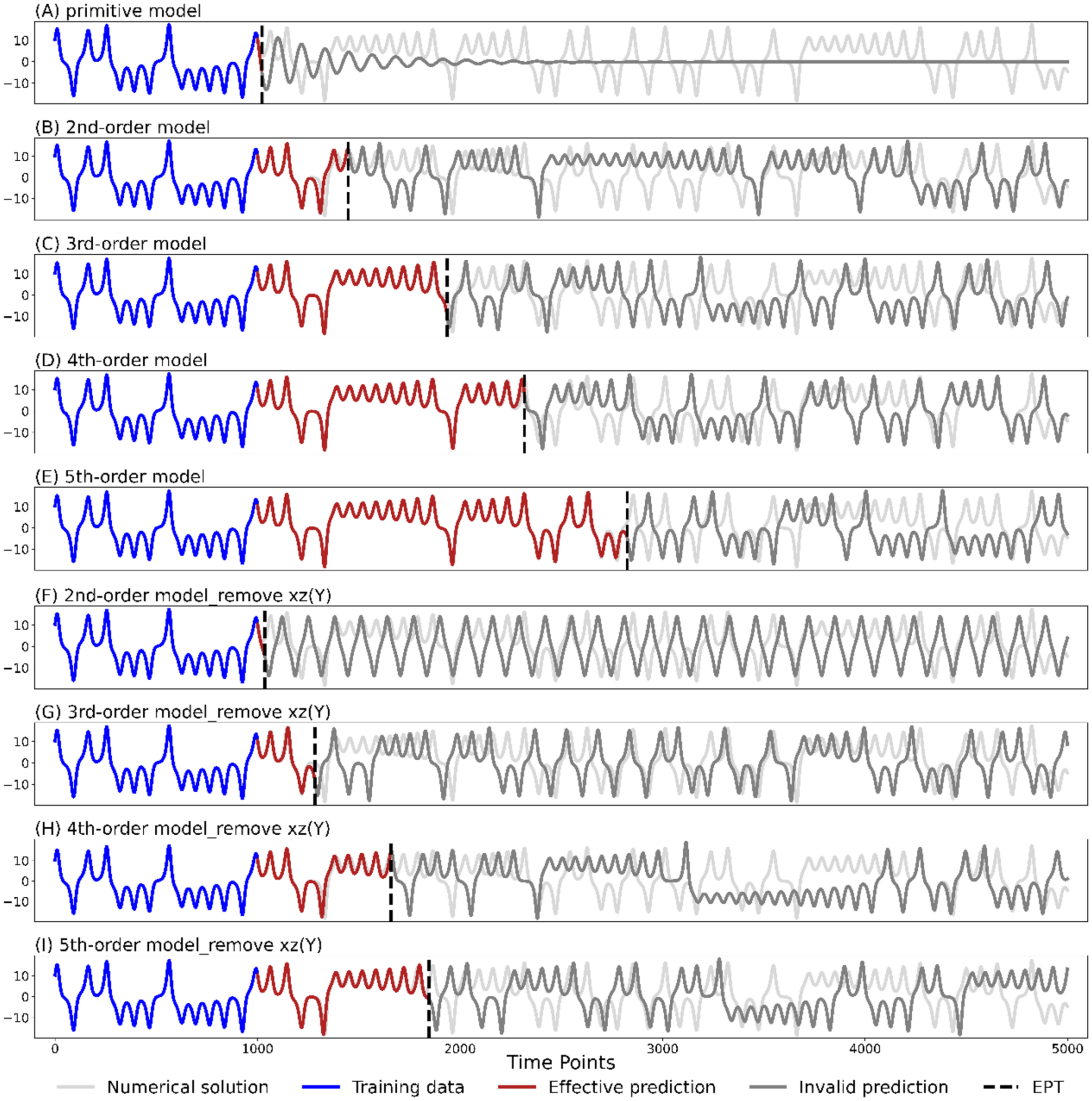
The roles of different layers in the DSDL model for the Lorenz system. (**A**–**E**) The prediction series of variable $x$ in the Lorenz system resulted from adding layer 1–5 into the DSDL model, respectively. (**F**–**I**) The prediction series of variable $x$ after removing one key variable from the second-order to fifth-order prediction model, respectively. The light grey line shows the numerical solution (true state), the blue line shows the training data set, the red line represents the effective predictions while the dark grey line represents the invalid predictions. The vertical black dashed line marks the EPT. Using a training set of 10^4^ time points, only the last 10^3^ time points are shown in this figure.

The prediction series of adding layer 1 is consistent with the linear model, which also converges quickly to a fixed point with basically no effective predictions (Fig. 4A). While the prediction series is immediately able to maintain the nonlinear characteristics and greatly improve the predictive ability after adding layer 2 (Fig. 4B). This indicates that layer 2, that is, key variables of second-order, plays a very important role in reconstructing dynamics of the Lorenz system and provides the necessary chaotic characteristics for the DSDL model.

#### Informativeness

Although adding layers 3– 5 cannot affect the chaotic characteristics of the DSDL model as much as layer 2, they will still change the evolution trajectories, improving prediction performance layer by layer (Fig. 4C– E). Therefore, the role of high-order key variables is likely to provide more information on nonlinear interactions among variables and add more constraints into the modulation of the DSDL model, so as to make model predictions follow the underlying evolution rules of the target variable as long as possible.

#### Robustness

Due to the difficulty in collecting all needful information of the target system in real-world modeling, we assume that a critical factor is unexpectedly missing from the prediction model. For example, after removing $xz$ from layer 2, the second-order model only exhibits a quasi-periodic evolution (Fig. 4F). However, the DSDL model can still provide a certain degree of accurate predictions after adding higher-order layers, demonstrating the robustness of the DSDL model (Fig. 4G – I).

## Discussion

In summary, we have proposed a new framework to make relative long-term accurate and transparent predictions of chaotic dynamical systems, and this DSDL method has been shown to be a successful scheme for dynamics-based deep learning. According to the embedding theorems, we can establish a prediction model based on the map between two kinds of reconstructed attractors. One is the delayed attractor reconstructed by the time-lagged coordinates of the target variable, and the other is the non-delayed attractor reconstructed by multiple key variables selected through CVSR method. The novelty of DSDL models, on the one hand, roots in a full exploitation of the nonlinear interactions among the multivariate time series data by constructing the multi-layer nonlinear network. On the other hand, using the CVSR method to select key variables that truly determine the evolution of the target variable, DSDL not only improves the model predictive capability, but also realizes the reduction of model complexity and improvement of model interpretability, that is to open the “black-box”. Notably, our DSDL model outperforms other existing dynamical and machine learning methods in four chaotic dynamical systems with different complexities.

However, we have to admit that the DSDL method still has certain limitations at present. In this study, we only focus on the prediction of ordinary differential equation systems, and we will continue to test the predictive performance of the DSDL model in partial differential equation systems. And we only focus on those chaotic dynamical systems whose equations are known. On this basis, we will further investigate those complex systems with uncertain structures and unknown equations. In addition, our study only considers data generated by noise-free numerical simulations, but noise is also inevitable in practical applications, thus the impact of noise on the DSDL model is also one of our focuses in the future. In this work, we simply set $\tau$ as one lag in the time series, and we still need to discuss the impact of time delay $\tau$ on the DSDL model in future work. Last but not least, Li and Chou^45^ proved the existence of the atmospheric attractor and the global analysis theory of climate system^46^ indicates that there exists a global attractor in the dynamical equations of climate, and any state of climate system will evolve into the global attractor as time increases. Therefore, this means that we may be able to apply the DSDL method to predict real-world systems in future work, but this requires more effort and validation.

## Methods

### Construction of multi-layer nonlinear network

Before describing the specific construction of the multi-layer nonlinear network in detail, we need to clarify some useful definitions. Suppose that there is a power function $x^{a} \left( {a = 0,1,2,3, \ldots } \right)$, its order is denoted as $O\left( {x^{a} } \right) = a$. On this basis, we can define a nonlinear monomial $F$ constructed by the product of power functions of primitive system variables, denoted as

1 $$F = F^{k} \left( {\varvec{X}} \right) = F^{k} \left( {x_{1} , \ldots ,x_{n} } \right) = \mathop \prod \limits_{i = 1}^{n} x_{i}^{{a_{i} }} ,$$

where $\mathop \sum \limits_{i = 1}^{n} a_{i} = k$, and the order of this monomial is

2 $$O\left( {F^{k} \left( {\varvec{X}} \right)} \right) = \mathop \sum \limits_{i = 1}^{n} a_{i} = k.$$

Therefore, we can eventually give the definition of the *i*th layer of the multi-layer nonlinear network ${\varvec{X}}_{{N_{{L_{i} }} }}^{{L_{i} }} , i \in \left[ {1,m} \right]$, that is, containing all *i*th-order monomials $F^{i} \left( {\varvec{X}} \right)$, which can be expressed as

3.1 $${\varvec{X}}_{{N_{{L_{1} }} }}^{{L_{1} }} = \mathop \cup \limits_{n} F^{1} \left( {\varvec{X}} \right) = {\varvec{X}},{ }$$

3.2 $${\varvec{X}}_{{N_{{L_{m} }} }}^{{L_{m} }} = \mathop \cup \limits_{n} F^{m} \left( {\varvec{X}} \right).{ }$$

What’s more, we need to further clarify some properties of the multi-layer network:

1. The number of nonlinear layers ($m$) varies with different chaotic dynamical systems, which will be determined by corresponding training/test data sets.
2. There is no intersection between two different nonlinear layers, which can be expressed as
  4 $${\varvec{X}}_{{N_{{L_{i} }} }}^{{L_{i} }} \cap {\varvec{X}}_{{N_{{L_{j} }} }}^{{L_{j} }} = \emptyset \left( {i,j \in \left[ {1,m} \right],i \ne j} \right).$$
3. Suppose that the target variable is $x_{k} \left( t \right)$, the key variables set of $x_{k} \left( t \right)$ selected from the *i*th layer is named as ${\varvec{X}}_{{K_{{L_{i} }} , x_{k} }}^{{L_{i} }} ,i \in \left[ {1,m} \right]$. Key variables selected in the previous layer will be input to the next layer and continue to join the selection procedure, denoted as,
  5.1 $${\varvec{X}}_{{K_{{L_{1} }} , x_{k} }}^{{L_{1} }} \subseteq {\varvec{X}}_{{N_{{L_{1} }} }}^{{L_{1} }} ,\user2{ }$$
  5.2 $${\varvec{X}}_{{K_{{L_{2} }} , x_{k} }}^{{L_{2} }} \subseteq {\varvec{X}}_{{N_{{L_{2} }} }}^{{L_{2} }} \cup {\varvec{X}}_{{K_{{L_{1} }} , x_{k} }}^{{L_{1} }} ,$$
  5.3 $${\varvec{X}}_{{K_{{L_{m} }} , x_{k} }}^{{L_{m} }} \subseteq {\varvec{X}}_{{N_{{L_{m} }} }}^{{L_{m} }} \cup {\varvec{X}}_{{K_{{L_{m - 1} }} , x_{k} }}^{{L_{m - 1} }} .$$

After all those procedures, we can achieve the final result of key variable set $\user2{ X}_{{K, x_{k} }} = {\varvec{X}}_{{K_{{L_{m} }} , x_{k} }}^{{L_{m} }}$.

### Selection method of key variables

After the construction of multi-layer nonlinear network, we need to select the key variables that play a decisive role in the time evolution of the target variable. Guo et al.^32^ proposed the cross-validation-based stepwise regression (CVSR) approach, which is a “forward” stepwise screening procedure to select the optimal predictors from the potential predictor set. The criteria for selecting key variables no longer rely on the fitting ability of the regression equation to be evaluated, but rather on the hindcast ability of the prediction model in cross validation. It employs *k*-fold cross validation to improve the robustness of selecting and avoid over-fitting effectively, and *k* in this work is equal to 10. The root-mean-square error between real data and cross-validation estimates is taken as the criterion to evaluate the performance of potential predictors.

### Assessments of model predictive capability

In order to quantify the performances of different models, we use the effective prediction time (EPT) to represent the model predictive capability, defined as the elapsed time before the corresponding prediction error $E\left( t \right)$ first exceeds an error threshold $\varepsilon$, and we have

6.1 $$E\left( t \right) = \left| {X\left( t \right) - \tilde{X}\left( t \right)} \right|,$$

6.2 $$EPT = Time\left[ {E\left( t \right) \le \varepsilon } \right],$$

where $\tilde{X}\left( t \right)$ represents the prediction series obtained by the model and $\varepsilon$ is equal to one standard deviation of the predicted time series $X\left( t \right)$ in this paper. The higher the EPT, the stronger the model predictive capability. Here, we denote the EPT in terms of model time units (MTUs), where 1 MTU $=$ 100 $\Delta t$. In order to make the results more robust, we use the mean EPT in assessing the model predictive capability, which is the average EPT of models in 100 different training/test sets. And we normalize the mean EPT by each attractor’s Lyapunov time.

### Supplementary Information

Supplementary Information.

## Data Availability

All data generated or analyzed during this study are included in this published article and its supplementary information files.
