# Supplementary material for: Interpretable predictions of chaotic dynamical systems using dynamical system deep learning
Source: Sci Rep. 2024 Feb 7;14:3143. doi: 10.1038/s41598-024-53169-y (PMC10850482; doi:10.1038/s41598-024-53169-y)

**Supporting Information for**

Interpretable predictions of chaotic dynamical systems using Dynamical System Deep Learning

Mingyu Wang^1^, Jianping Li ^1,2*^

1. Frontiers Science Center for Deep Ocean Multi-spheres and Earth System (FDOMES)/Key Laboratory of Physical Oceanography/Academy of Future Ocean, Ocean University of China, Qingdao 266100, China
2. Laoshan Laboratory, Qingdao 266237, China

*Correspondence to: Jianping Li (ljp@ouc.edu.cn)

**This PDF file includes:**

Supporting Information Text

SI References

Figures S1 to S6

Tables S1

Supporting Information Text

**Data**

**Lorenz System**

The Lorenz system is the first and the most representative chaotic dynamical system, which represents chaotic characteristics of the atmosphere. The state variables $x$, $y$, and $z$ represent measures of fluid velocity and the spatial temperature distribution in the fluid layer under gravity^1,2^. The 3-variable Lorenz system is

$$\left\{ \begin{aligned} &\frac{dx}{dt}=\sigma\left( y-x \right) \\ &\frac{dy}{dt}=rx-y-xz \\ &\frac{dz}{dt}=xy-bz \end{aligned} \right. , (1)$$

where $\sigma=10, r=28$, and $b=8/3$, for which the well-known butterfly attractor exists. Using a fourth-order Runge-Kutta scheme with a time step $\Delta t=0.01$ dimensionless time units (TUs), we integrate forward for ${10}^{7}$ steps starting from $\left( x, y, z \right)=$(0, 1, 0) to generate a large dataset for training and testing, and we discard the first 5000 time points to ensure the chaos of time series.

**Hyperchaotic Lorenz System**

The hyperchaotic system, which has more than one positive Lyapunov exponent, has increasingly aroused the interest due to its great potential in technological applications over many fields, such as secure communication, lasers, and so on^3^. Li et al.^4^ proposed a new hyperchaotic system through introducing an additional state variable into the 3-variable Lorenz system. The equations that describe the 4-variable hyperchaotic Lorenz system are

$$\left\{ \begin{aligned} &\frac{dx_{1}}{dt}=a\left( x_{2}-x_{1} \right) \\ &\frac{dx_{2}}{dt}=bx_{1}+cx_{2}-x_{1}x_{3}+x_{4} \\ &\frac{dx_{3}}{dt}=-dx_{3}+x_{1}x_{2} \\ &\frac{dx_{4}}{dt}={-kx}_{1} \end{aligned} \right. , (2)$$

where $x_{i}(i=1,2,3,4)$ are state variables. Here, taking $a=35, b=7, c=12, d=3,$ and $k=5$, the hyperchaotic attractor exists. Using a fourth-order Runge-Kutta scheme with a time step $\Delta t=0.01$ dimensionless time units (TUs), we integrate forward for ${10}^{7}$ steps starting from $\left( x_{1}, x_{2}, x_{3},x_{4} \right)=$(0, 1, 0, 0) to generate a large dataset for training and testing, and we discard the first 5000 time points to ensure the chaos of time series.

**Conceptual Ocean-Atmosphere Coupled Lorenz System**

Since the Lorenz system cannot reflect the coupling processes between the atmosphere and the ocean^5^, Zhang et al.^6^ add a slowly changing variable *w*, which is coupled with the 3-variable Lorenz system to simulate the interaction of the fast system with the slow upper ocean, called 4-variable coupled model (4VCM). At the same time, Zhang^7^ adds the deep ocean pycnocline ($\eta$) on the 4VCM to simulate the fundamental features of the real climate system, called 5VCM. Here we take the 5VCM as a conceptual ocean-atmosphere coupled model that describes typical interactions of a synoptic-scale atmosphere with a seasonally−interannually varying upper ocean as well as a deep ocean that varies on decadal timescales. Here, the conceptual ocean-atmosphere coupled system takes the form as

$$\left\{ \begin{aligned} &\frac{dx}{dt}=\sigma\left( y-x \right) \\ &\frac{dy}{dt}=(1+c_{1}w)\kappa x-y-xz \\ &\frac{dz}{dt}=xy-bz \\ &O_{m}\frac{dw}{dt}=c_{2}y+c_{3}\eta+c_{4}w\eta-O_{d}w+S_{m}+S_{s}\cos(2\pi t/S_{pd}) \\ &\Gamma\frac{d\eta}{dt}=c_{5}w+c_{6}w\eta-O_{d}\eta\end{aligned} \right. , (3)$$

which includes 5 state variables ($x, y, z$ for the atmosphere, $w$ for the upper ocean, and $\eta$ for the deep ocean pycnocline), with the values of 15 parameters described above, $(\sigma, \kappa, b, c_{1}, c_{2}, O_{m}, O_{d}, S_{m}, S_{s}, S_{pd}, \Gamma, c_{3}, c_{4}, c_{5}, c_{6})=$(9.95, 28, 8/3, ${10}^{-1}$, 1, 10, 1, 10, 1, 10, 100, ${10}^{-2}$, ${10}^{-2}$, 1, ${10}^{-3}$). Using a fourth-order Runge-Kutta scheme with a time step $\Delta t=0.01$ dimensionless time units (TUs), we integrate forward for ${10}^{7}$ steps starting from$\left( x, y, z, w, \eta\right)=$(0, 1, 0, 0, 0) to generate a large dataset for training and testing, and we discard the first 5000 time points to ensure the chaos of time series.

**Mackey-Glass equation**

The Mackey-Glass equation^8^ is the nonlinear time delay differential equation

$$\dot{x}(t)=-bx(t)+\frac{ax(t-\tau)}{1+x^{c}(t-\tau)} , (4)$$

where $a=0.2, b=0.1, c=10,$ and$\tau=17$. Here, an input time window with time delays ($t, t-4, t-8,t-12,t-16)$ is used for different approaches. Using a fourth-order Runge-Kutta scheme with a time step $\Delta t=0.1$ dimensionless time units (TUs), we integrate forward for ${10}^{7}$ steps to generate a large dataset for training and testing, and we discard the first 5000 time points to ensure the chaos of time series.

**Training sets and test sets**

Suppose we need to start predicting from the point $N$ in the observed time series (numerical solution) of one system, then the training set with sufficient length is ${10}^{4}$ sequential samples before $N$ and the test set is the next ${5\times10}^{3}$ sequential samples after $N$. Starting from 100 random points, we have randomly chosen 100 such training/test sets in all three systems.

**Other existing dynamical and machine learning methods used for comparison**

We compare DSDL with the following seven existing dynamical and machine learning methods, IDE^9^: Inverse Delayed Embedding method, which is proposed as an inverse ﻿implementation of ﻿the delayed embedding reconstruction to provide a new way to predict the target system; SVE^10^: the classic Single Variable Embedding method, making predictions only based on the time series of the target variable; AR^11^: a traditional Auto-Regressive model for predicting time series; VAR^12^: a Vector Auto-Regressive model, which is a multi-variable model capturing the pairwise relationships among all variables; Lasso^13^: the Lasso procedure is used to estimate the parameters of AR and make predictions; SVR^14^: Support Vector Regression method, which uses support vector machine to fit curves and perform regression analysis; LSTM^15^: A famous neural network which is widely used in the field of time series analysis; ResNet^16^: Residual Network, one of the most popular deep learning neural network, which can effectively solve the degradation problem and gradient disappearance; NG-RC^17^: Next generation reservoir computing, which demonstrates that nonlinear vector autoregression excels at reservoir computing benchmark tasks and requires even shorter training data sets and training time, heralding the next generation of reservoir computing.

**Improvements by selecting key variables**

The number of layers for the multi-layer nonlinear network varies with different dynamical systems in the light of their diverse chaotic characteristics. For the Lorenz system, predictive abilities of DSDL models are the highest when five nonlinear layers have been added, namely a fifth-order model (Figure S3A). After that, adding more layers of higher-order will be obstacles to reconstruct dynamics of the target system. With suitable number of nonlinear layers, the DSDL can further extract key variables that are truly essential for the temporal evolution of the target variable and remove the irrelevant information. To illustrate the roles of selecting key variables, the prediction abilities of models built with all variables in the fifth-order model or just key variables selected from the fifth-order model are compared in 100 different training/test sets of the Lorenz system. On average, the predictive abilities of key-variable models are better than that of all-variable models (Figure S4A); separately, the EPTs of key-variable models are better than or equal to that of all-variable models in 95% of the 100 sets (Figure S4B).

Using a fifth-order model for the hyperchaotic Lorenz system (Figure S3B) and a fourth-order model for the coupled Lorenz system (Figure S3C), the similar results are shown in both systems (Figures S5, 6). On the one hand, selecting key variables robustly increases the predictive capability of the DSDL model. On the other hand, this selection process excludes factors that have little or no effect on the temporal evolution of the target variable, which allows us to effectively reduce the dimensionality of the prediction model. Furthermore, the selection of key variables makes it hopefully easier for us to explain the DSDL prediction model.

**SI References**

1. Lorenz, E. N. Deterministic nonperiodic flow. *J Atmos Sci* 20, 130-141 (1963).
2. Ding, R. Q. & Li, J. P. Relationships between the limit of predictability and initial error in the uncoupled and coupled Lorenz models. *Adv. Atmos. Sci.* 29, 1078–1088 (2012).
3. Wang, F. Q. & Liu, C. X. Synchronization of hyperchaotic Lorenz system based on passive control. *Chinese Phys.* 15, 1971-1975 (2006).
4. Li, Y. X., Wallace, K. S. & Chen, G. R. Hyperchaos evolved from the generalized Lorenz equation. *Int. J. Circuit Theory Appl.* 33, 235-251 (2005).
5. Zhao, H. R., Zhang, S. Q., Li, J. P. & Ma, Y. W. A study of predictability of coupled ocean–atmosphere system using attractor radius and global attractor radius. *Clim. Dyn.* 56, 1317-1334 (2021).
6. Zhang, S. Q., Liu, Z., Rosati, A. & Delworth, T. A study of enhancive parameter correction with coupled data assimilation for climate estimation and prediction using a simple coupled model. *Tellus A* 64, 10963 (2011).
7. Zhang, S. Q. A Study of impacts of coupled model initial shocks and state–parameter optimization on climate predictions using a simple pycnocline prediction model. *J. Clim.* 23, 6210-6226 (2011).
8. Mackey, M. & Glass, L. Oscillation and Chaos in Physiological Control Systems. *Science* **197** (1977).
9. Ma, H. F. et al. Predicting time series from short-term high-dimensional data. *Int J Bifurcat Chaos* **24**, 143003 (2014).
10. Farmer, J. D. & Sidorowich, J. J. Predicting chaotic time series. *Phys. Rev. Lett.* **59**, 845 (1987).
11. Kishida, K. Autoregressive model analysis and decay ratio. *Ann Nucl Energy* 17, 157-160 (1990).
12. Ramirez-Beltran, N. D. A vector autoregressive model to predict hurricane tracks. *INT J SYST SCI* 27, 1-10 (1996).
13. Nardi, Y. & Rinaldo, A. Autoregressive process modeling via the Lasso procedure. *J Multivariate Anal* 102, 528-549 (2011).
14. Trafalis, T. B. & Ince, H. Support vector machine for regression and applications to financial forecasting. *Neural Computing: New Challenges and Perspectives for the New Millennium* 6, 348-353 (2000).
15. Hochreiter, S. & Schmidhuber, J. Long short-term memory. *Neural Comput* 9, 1735–1780 (1997).
16. He, K. M., Zhang, X. Y., Ren, S. Q. & Sun, J. Deep residual learning for image recognition. *IEEE Conference on Computer Vision and Pattern Recognition* 770-778 (2016).
17. Gauthier, D. J. & Bollt, E., Griffith, A. et al. Next generation reservoir computing. *Nat Commun* 12, 5564 (2021).

Supplementary Figures and Tables


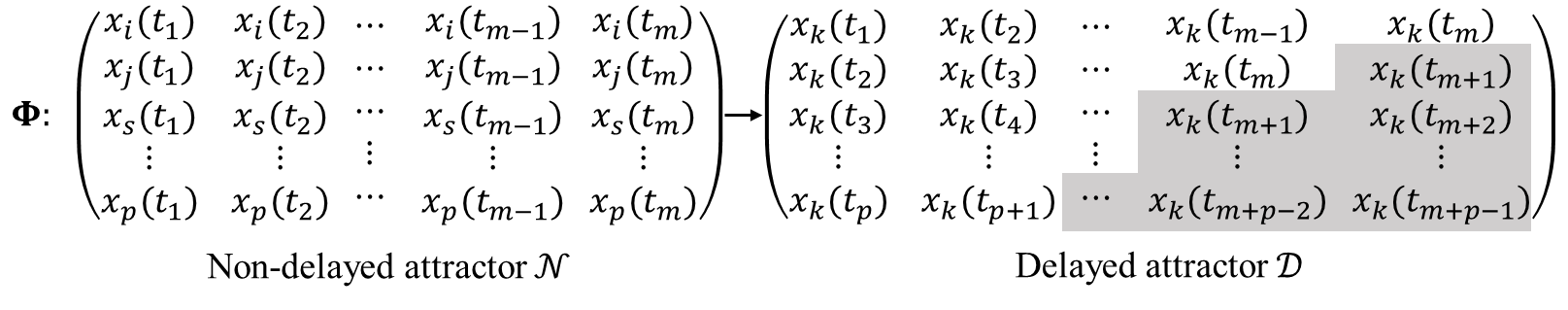


**Figure S1 | Matrix form of the diffeomorphism map Φ between** **the delayed attractor** $\mathcal{D}$ **and the non-delayed attractor** $\mathcal{N}$. All elements of the non-delayed attractor constructed by multiple key variables on the left-hand-side and the unshaded elements of the delayed attractor on the right-hand-side are available from the observed data, while the shaded variables of $x_{k}$ on the right-hand-side $x_{k}\left( t_{m+1} \right),\ldots, x_{k}\left( t_{m+p-1} \right)$ are values to be predicted. The second component of the right-hand-side plays as a one-step predictor, so a straightforward way for multi-step prediction is to obtain the one-step predictor for each variable of the system and use it iteratively. The second way for multi-step prediction is to use different components of **Φ** for the target variable $x_{k}$. Specifically, after the one-step prediction $\tilde{x_{k}}\left( t_{m+1} \right)$ using the second component, the third component of **Φ** could be further trained as a two-step predictor to get $\tilde{x_{k}}\left( t_{m+2} \right)$, which could be conducted continuously.

**
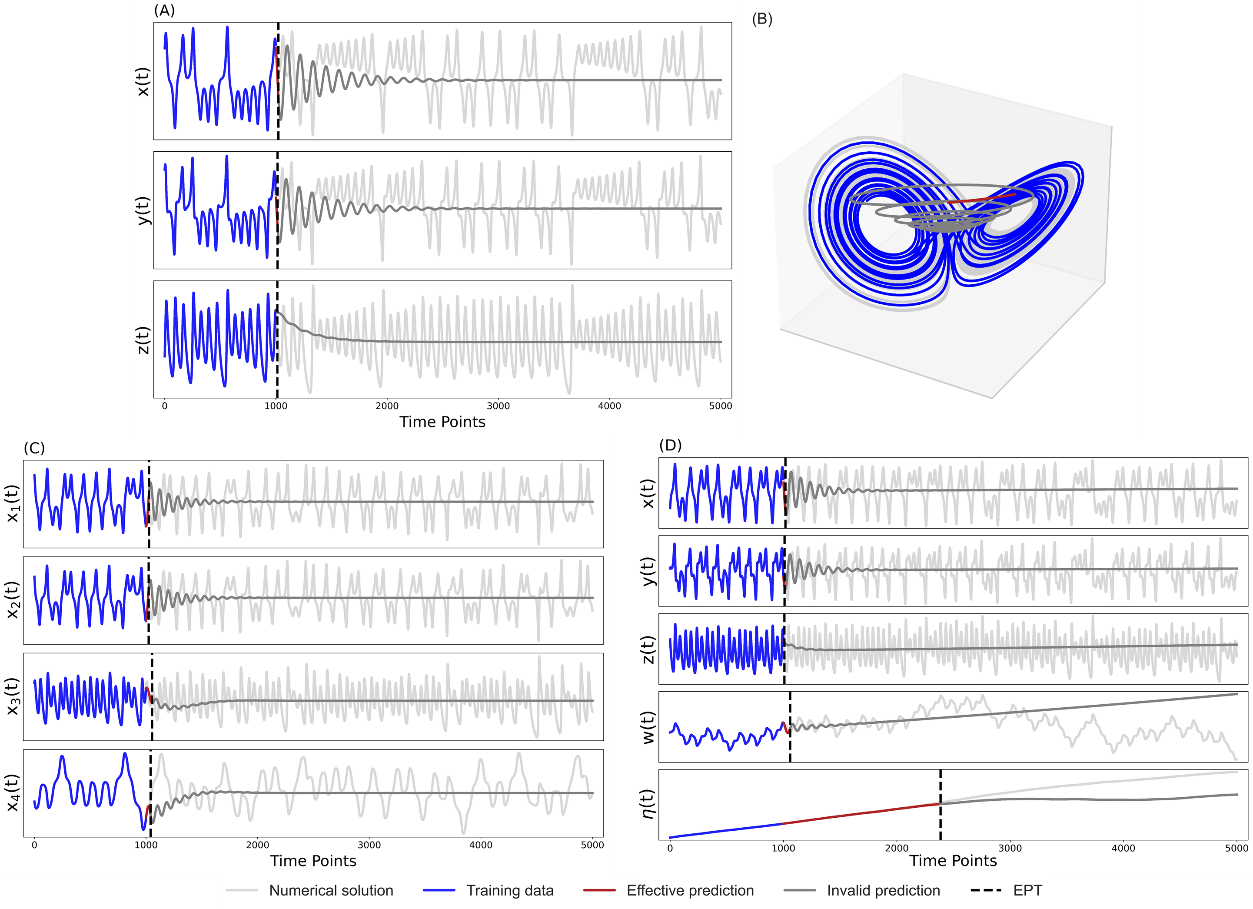
**

**Figure S2 | Prediction results of linear models in three different chaotic dynamical systems.** (A) The prediction series of the Lorenz system. The light grey line shows the numerical solutions (true state), the blue line shows the training set, the red line represents the effective predictions and the dark grey line represents the invalid predictions in the corresponding test set. The vertical black dashed line marks the effective prediction time (EPT). Using a training set of 10^4^ time points, only the last 10^3^ time points are shown in this figure. (B) The prediction trajectory of the Lorenz system. (C) Same as (A), but for the hyperchaotic Lorenz system. (D) Same as (A), but for the conceptual ocean-atmosphere coupled Lorenz system.


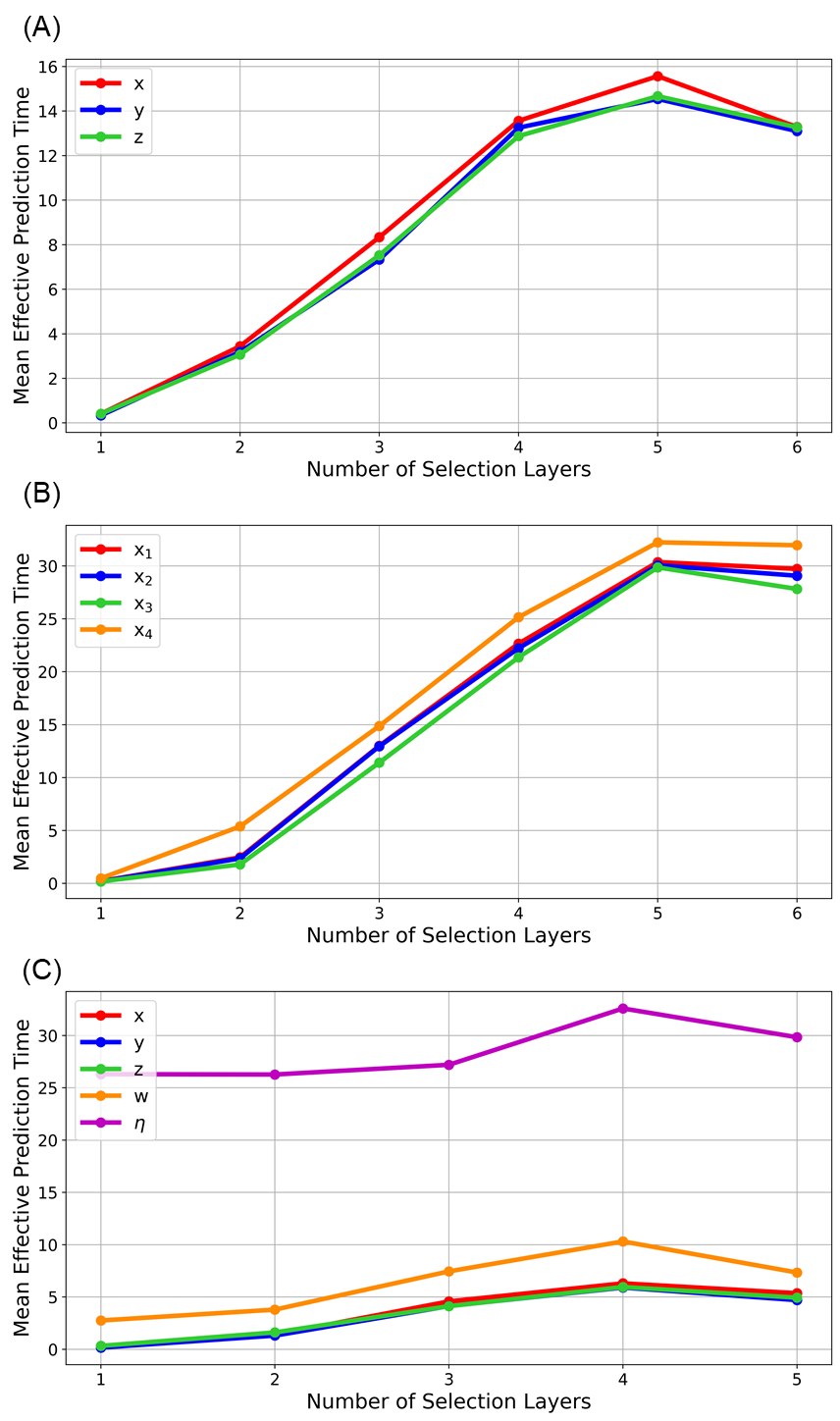


Figure S3 | The effect of different numbers of nonlinear layers on the model predictive capability in three different systems. (A) The result of the Lorenz system. The red line represents the variable $\boldsymbol{x}$, the blue line represents the variable $\boldsymbol{y}$ and the green line represents the variable $\boldsymbol{z}$ of the Lorenz system. Mean EPT is obtained by 100 different training/test sets to quantify the model predictive capability. (B) Same as (A), but for the hyperchaotic Lorenz system. (C) Same as (A), but for the conceptual coupled Lorenz system.


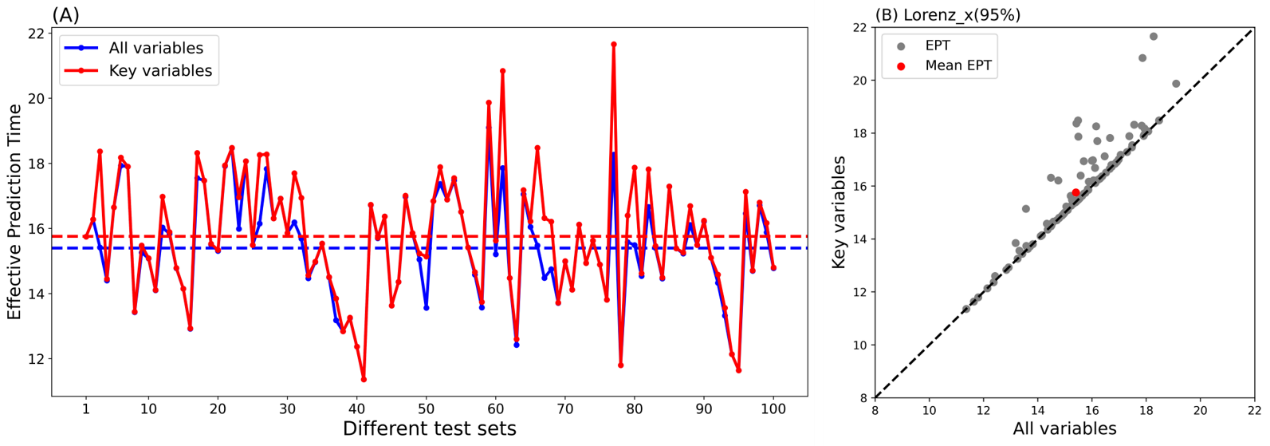
Figure S4 | Comparing the predictive capabilities of DSDL models built with all variables or key variables in the Lorenz system. (A) Predictive capabilities of two kinds of models in 100 different training/test sets. The blue horizontal dashed line shows the mean EPT of 100 data sets using models with all variables, while the red line shows the result of models with key variables. (B) Scatter plot comparing the EPTs of models using all variables or key variables. The grey dots represent the 100 pairs of EPTs, and the red dot represents the mean EPT dot pair. 95% represents 95 out of 100 sets where the model predictive capabilities using key variables are greater than or equal to that using all variables.


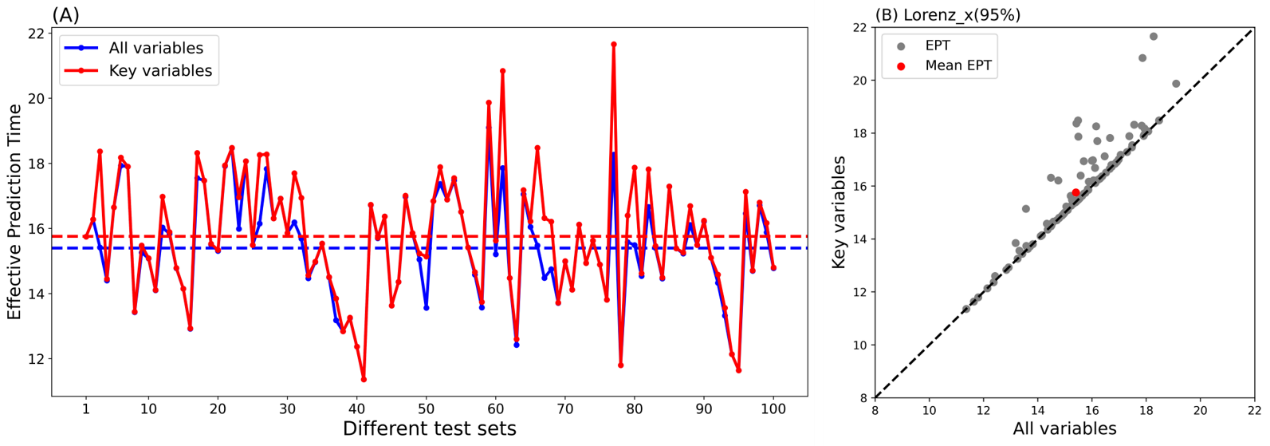


Figure S5 | Same as Figure S4, but for the hyperchaotic Lorenz system.


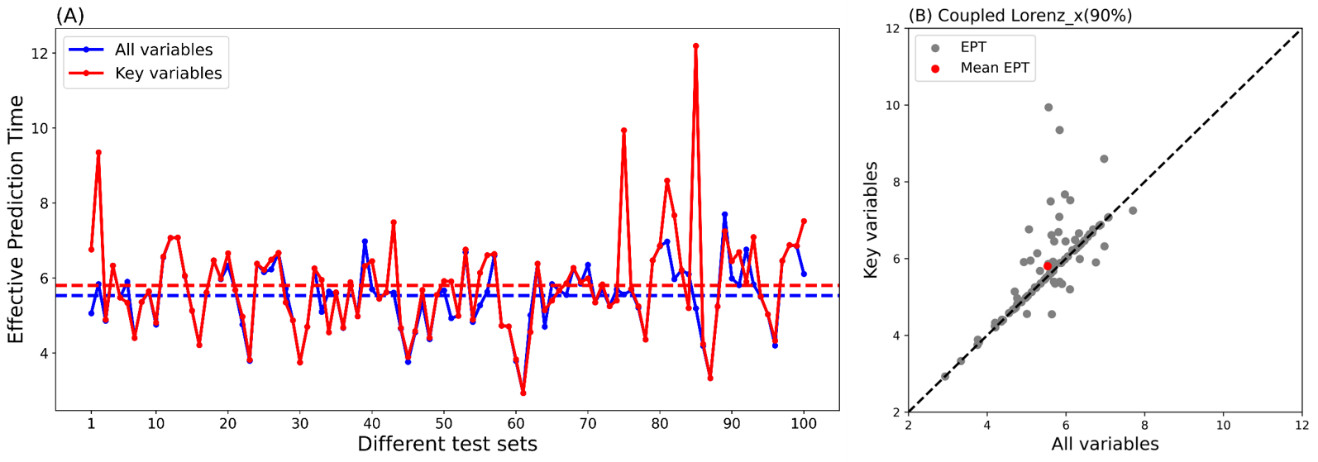


Figure S6 | Same as Figure S4, but for the coupled Lorenz system.

Table S1 | Key variables of different nonlinear layers for the Lorenz system. The blue variables represent the terms of the Lorenz equations, and the grey variables indicate that the key variables of the current layer are not selected into the next layer. This is only the result using one training set.


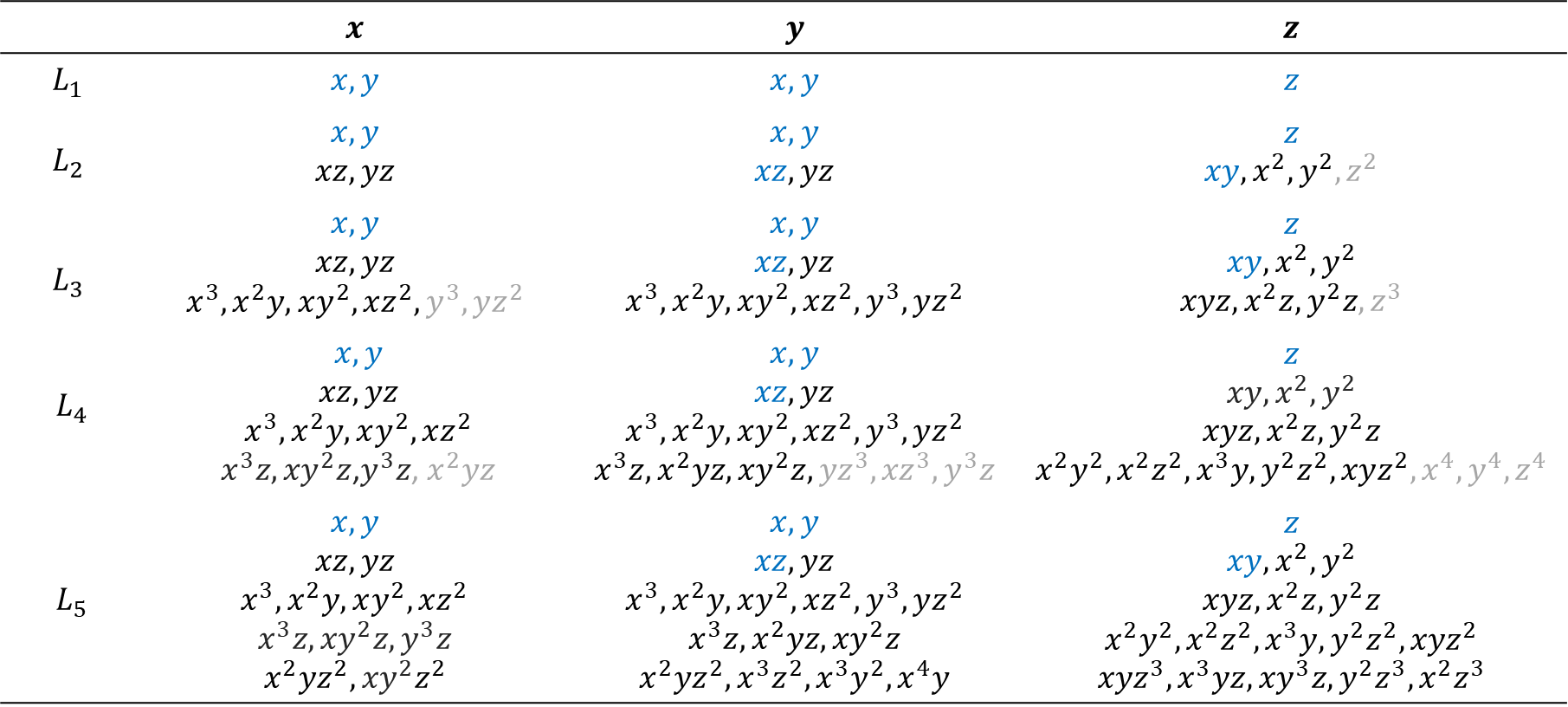

Supplement: Supplementary file 1 — Supplementary Information. [file 41598_2024_53169_MOESM1_ESM.docx]
